# Structure and stability of the coral microbiome in space and time

**DOI:** 10.1038/s41598-019-43268-6

**Published:** 2019-05-01

**Authors:** Courtney M. Dunphy, Tarik C. Gouhier, Nathaniel D. Chu, Steven V. Vollmer

**Affiliations:** 1 Marine Science Center, Northeastern University, 430 Nahant Road, Nahant, MA 01908 USA; 2 Microbiology Graduate Program, Massachusetts Institute of Technology, Cambridge, Massachusetts, 02139 USA

**Keywords:** Microbial ecology, Community ecology, Microbial ecology, Population dynamics

## Abstract

Although it is well established that the microbial communities inhabiting corals perform key functions that promote the health and persistence of their hosts, little is known about their spatial structure and temporal stability. We examined the natural variability of microbial communities associated with six Caribbean coral species from three genera at four reef sites over one year. We identified differences in microbial community composition between coral genera and species that persisted across space and time, suggesting that local host identity likely plays a dominant role in structuring the microbiome. However, we found that microbial community dissimilarity increased with geographical distance, which indicates that regional processes such as dispersal limitation and spatiotemporal environmental heterogeneity also influence microbial community composition. In addition, network analysis revealed that the strength of host identity varied across coral host genera, with species from the genus *Acropora* having the most influence over their microbial community. Overall, our results demonstrate that despite high levels of microbial diversity, coral species are characterized by signature microbiomes that are stable in both space and time.

## Introduction

Reef-building corals live in association with endosymbiotic algae from the genus *Symbiodinium* and a diverse array of bacteria^1–3^. The alliance between the coral animal, endosymbiotic algae, and microorganisms, termed the holobiont^4^, has allowed corals to colonize many diverse marine habitats and form massive reef structures^4,5^. Although the coral-algal symbiosis is well characterized, less is known about the factors that control coral-bacterial interactions. Recent evidence linking coral diseases to bacterial pathogens^6–11^ suggests that quantifying variation in the composition of healthy microbiomes across coral species, space and time is critical in order to both understand the stability of host-microbial interactions and potentially predict disease outbreaks.

Generating baseline knowledge about the stability of host-microbial interactions is key in understanding the coral-microbial symbiosis, as breakdowns in this relationship (i.e. disease outbreaks) have caused dramatic declines in coral cover worldwide, shifting reefs towards algae-dominated systems^2,5,12,13^. However, despite increased disease frequency and severity^5,14^, linking coral diseases to their putative pathogens remains a difficult task^2^, partially because of high coral microbial diversity and our limited understanding of the natural dynamics of coral microbiomes. Although recent studies have begun to characterize the microbiome of healthy corals^15–18^, the underlying mechanisms that determine microbial community structure remain largely unknown.

The difficulty in elucidating the mechanisms behind coral-microbial interactions is mainly attributable to (i) the relatively high diversity of coral microbes (but see McDevitt-Irwin^19^), (ii) the small fraction of culturable microbes, and (iii) the lack of a foundational understanding of the microbial community in space and time. Rohwer *et al*.^4,20^ were the first to apply culture-independent, DNA-based techniques to study coral bacteria. They demonstrated that three Caribbean corals had a diverse bacterial community, including a majority of novel species. Since then, coral microbial sequencing efforts have continued to provide evidence that coral microbial communities appear to be host species-specific, and differ from the microbes dominating the surrounding reef water^4,15,18,21,22^. With advances in microbial metagenomic sequencing and increased interest in coral-microbial symbiosis, there are now varying levels of insight regarding microbial abundance, specificity, spatiotemporal structure, roles and interactions, and modes of acquisition^23–27^.

Recently, coral-microbiome studies have shifted their focus from examining the whole microbiome of a coral species to a core microbial framework^28^, which aims to identify critical microbes based on their persistence within a host^29^. Hernandez-Agreda^30^ proposed partitioning the coral microbiome into three separate components: an environmentally responsive community (predominantly transient microbes linked to abiotic factors), a resident or individual microbiome, and a core microbiome (small group of highly persistent OTUs). Additionally, they determined that a coral’s resident microbiome contained less than 3% of the total bacterial phylotypes associated with all individuals of that species. Furthermore, despite being exposed to different stressors (nutrient pollution and herbivory), Zaneveld *et al*.^31^ described multiple levels (95%, 90%, 75%) of core microbial membership in three coral genera (*Porites, Siderastrea, and Agaricia*) across three years of repeated sampling of the same individuals. Overall, the core microbiome framework provides a way of identifying and examining potentially important microbes within the coral microbial community based on their persistent association to a coral group^29^.

Although focusing on the core microbiome is likely to yield key insights about the structure of host-microbial associations in space and time, linking these patterns to their underlying mechanisms is crucial for predicting the health, functioning and persistence of these important ecological systems. Host selection, environmental filtering, microbial dispersal limitation, and microbial species interactions have all been suggested as key drivers of host-microbial composition in space and time (reviewed by Costello *et al*.)^32^. For example, the ability of corals to acquire new symbionts in order to mitigate the adverse effects of environmental stresses has led to the development of the coral probiotic hypothesis^5,33^. This hypothesis posits that corals can shuffle their holobiont by selecting strains of dinoflagellates and microbes in order to promote the growth and persistence of the host under shifting environmental conditions^5^. One mechanism by which such host selection can occur is through the secretion of antibiotic compounds via the coral mucus layer that target non-beneficial or pathogenic microbes^34^. Indeed, multiple studies demonstrate selective antimicrobial production against fungus, Gram-positive bacteria, and known coral pathogens^35–37^. Even in the absence of host selection, the community structure of the host’s microbiome can aid in preserving a stable microbial community. For example, in an assay designed to differentiate between “visitor” or transient and “resident” bacteria, Ritchie^34^ found that antibiotic production by resident bacteria in the coral mucus was significantly higher than that of visitors. This suggests that under normal conditions, resident microbes may play a critical role in limiting the abundances of pathogenic microbes. More recently, a long-term study found that outbreaks of Proteobacteria opportunists were more common when Actinobacteria were in low abundance on corals, suggesting that antibiotic producing Actinobacteria are important for suppressing opportunists^31^.

In addition to local host identity and microbial competition, regional processes such as microbial dispersal and environmental heterogeneity can play an important role in structuring host-microbial systems^38^. Extrinsic factors such as reef habitat, nutrients and temperature (which also differ across locations and seasons), have been found to significantly affect the specificity of bacterial-coral associations^39–41^. Additionally, local environmental conditions are predicted to select for specific metabolic pathways in microorganisms that play an important role in coral health. For example, Kelly *et al*.^27^ found that microbial community metabolic potential was most strongly correlated with geographic location, suggesting that microbial community composition is not entirely dependent upon intrinsic factors (e.g., host identity and microbial interactions). Although microorganisms are prevalent throughout the ocean and evidence suggests that corals separated by thousands of kilometers can share a core microbiome^17,42^, dispersal limitation could still play an important role in determining the distribution of microbes by preventing them from reaching suitable hosts^43^. Overall, resolving the relative importance of host identity, microbial competition, microbial dispersal, and environmental heterogeneity is critical for understanding the stability of host-microbial systems.

Previous studies analyzing the natural variability of coral microbiomes indicate that both the coral host and the environment shape microbial community composition. Sunagawa *et al*.^15^ showed that bacterial communities clustered by coral species and that closely related corals had similar microbiomes. More recently, Chu & Vollmer^18^ detected a stable and unique set of bacterial phylotypes (core microbiome) associated with six Caribbean coral species. This suggests that the coral host was the strongest driver of coral microbiome composition across space and time and also indicates specific and divergent niches for microbial species. While their study focused on advancing our understanding of how coral microbiomes change over time and space, we sought to expand on their findings by focusing on the drivers of microbial community differences across spatiotemporal scales, the likely influence of the environment on community composition, and variation in the strength of phylosymbiosis across coral hosts.

We applied a combination of multivariate analyses and network modeling to coral-associated bacterial community data collected by Chu & Vollmer^18^ from three coral genera (*Acropora, Porites*, and *Diploria*), each containing two related species [*Acropora cervicornis*, *A. palmata*, *Porites astreoides*, *P. furcata*, *Diploria labyrinthinformis*, and *D*. (*Pseudodiploria*) *strigosa*], and sampled at multiple sites near Bocas del Toro, Panama, at three time points over one year. Our analyses identified consistent differences in microbial community composition between coral genera and species that persisted in space and time, suggesting that host identity plays a more important role in structuring coral microbiomes than spatiotemporal environmental heterogeneity or dispersal. However, our network analyses revealed that the strength of host identity varied significantly across coral species, with *Acropora* hosts being weakly connected to other corals’ microbiomes compared to either *Diploria* or *Porites* (i.e., *Acropora* corals were more similar to one another and different than other coral genera). Overall, our results suggest that local processes such as host phylogeny and microbial competition play an important role in stabilizing the coral microbiome across temporal and spatial scales.

## Results

### Distribution of microbial classes and core classes across coral hosts

**Figure 1:**
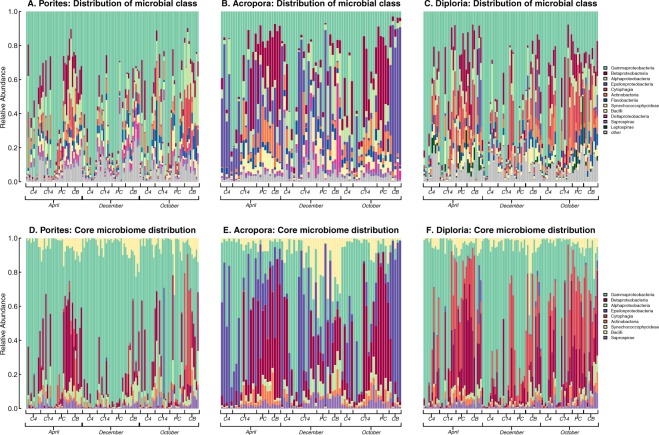
Prevalence and distribution of microbial classes (**A**) Porites, (**B**) Acropora and (**C**) Diploria and core microbiomes (**D**) Porites, (**E**) Acropora and (**F**) Diporia associated with coral genera *Porites, Acropora*, and *Diploria* across all sites and time points. Range bars on the x-axis show all samples collected within each time point.

To determine the dominant bacterial phylotypes in these communities across host, site, and time, we first analyzed the top proportionally represented microbes in the whole community data. Our samples included microbiomes of 100 coral individuals (hosts) from three highly abundant Caribbean coral genera across four reef sites at three time points over one year and found that overall Gammaproteobacteria phylotypes were the most dominant across all coral genera (relative abundances: *Porites*: 0.59, *Diploria*: 0.49, *Acropora*: 0.32; Fig. 1A–C). In the *Porites* samples, site Casa Blanca (CB) showed an overall higher amount of diversity across all time points (Fig. 1A). Temporally, Gammaproteobacteria phylotypes were more dominant in April and December than in October (relative abundances: April: 0.66, December: 0.64, and October: 0.43). Additionally, *Acropora* samples showed the highest relative abundances of phylotypes from Epsilonproteobacteria (*Acropora*: 0.290, *Diploria*: 0.009, and *Porites*: 0.004). Interestingly, within class Epsilonproteobacteria are Campylobacteraceae, these microbes have previously been described as being associated with white band disease in *Acropora cervicornis*^11^. Furthermore, Endozoicomonaceae (class Gammaproteobacteria) consistently showed greater abundances on *Acropora* and *Porites* across multiple sites and times (Supporting Information, Fig. S1). Recent studies of the genus *Endozoicomonas* have received considerable attention, as they appear to be extremely flexible symbionts and a putative beneficial bacteria associated with the health of corals worldwide^16,17,41,44–46^.

To determine whether each coral genus contained a unique core microbiome (defined as when a bacterial phylotype is present in >95% of individual coral genera samples within the study), we identified OTUs by presence or absence and calculated OTUs with the highest persistence across hosts within a coral genus. Abundances of identified core OTUs were used to determine individual OTU relative abundance across all hosts (grouped by taxonomic class; Fig. 1D–F). We found that although the three coral genera shared the same small group of highly persistent bacterial classes (with the exception of *Acropora* containing Bacilli within its core), their relative abundances differed considerably between coral genera. The core microbiome of *Porites* was dominated by phylotypes from Gamma-, Betaproteobacteria, and Synechococcophycideae (relative abundances: 0.70, 0.13, and 0.05, respectively; Fig. 1D). The *Acropora* core microbiome was dominated by Gamma-, Epsilon- and Betaproteobacteria (relative abundances: 0.31, 0.27, and 0.24, respectively; Fig. 1E). Interestingly, high persistence (>95%) and abundances of two phylotypes of Campylobacteriales (class Epsilonproteobacteria) was found across all samples of *Acropora* which, as mentioned above, have been connected to white band disease in *Acropora cervicornis*^11^. Additionally, *Acropora* core microbiomes showed the highest relative abundances of Endozoicomonaceae (0.22), followed by *Porites* (0.17) and *Diploria* (0.04). *Diploria’s* core microbiome was dominated by Gammaproteobacteria, Cytophagia, and Betaproteobacteria (relative abundances:0.52, 0.18, and 0.18, respectively; Fig. 1F).

### Multivariate analyses

Utilizing the full resolution of the data (OTUs), we performed a suite of multivariate analyses to test for differences among the total bacterial communities across coral hosts, sites and times. Additionally, we wanted to determine the independent and joint effects of coral host (at the genus vs. species level), spatial environmental variation (by comparing different sites), temporal environmental variation (by comparing different times), and spatiotemporal environmental variation (the two-way interaction between sites and times).

**Figure 2:**
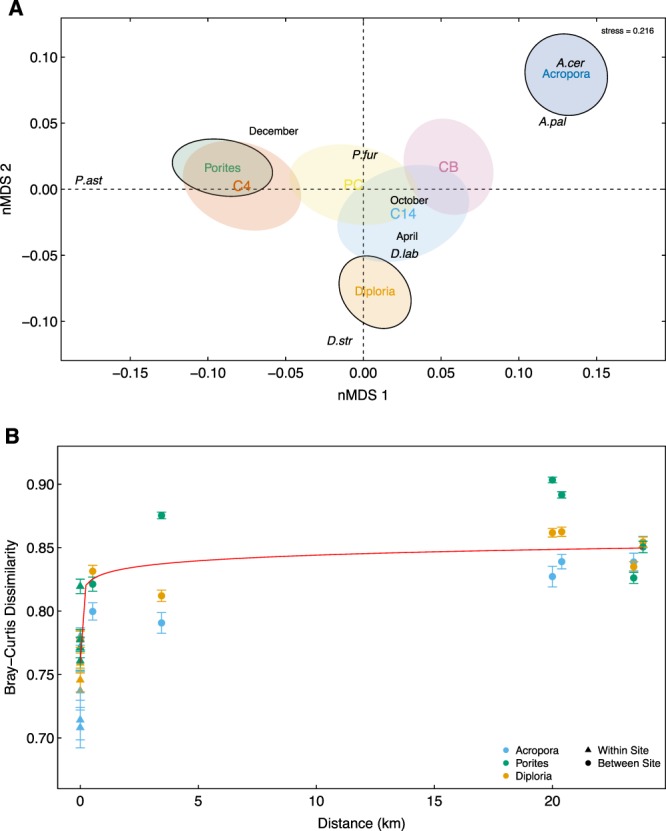
(**A**) Non-metric Multidimensional Scaling (nMDS) analysis based on Bray-Curtis dissimilarity between the bacterial communities of the coral samples. Labels: species and time; Ellipses for genera and site. (**B**) Mean (+/−s.e.) Bray-Curtis dissimilarity of microbial communities associated with each coral genus as a function of the geographical distance that separates them. The red trend line represents the significant positive relationship between Bray-Curtis dissimilarity and geographical distance obtained via ANCOVA (*p*-value = 0.0009). Both Bray-Curtis dissimilarity and geographical distance were log-transformed prior to conducting ANCOVA.

Ordination analysis of the microbial communities showed strong differences in total community composition between coral hosts, with no overlap between the 95% confidence ellipses representing each coral genus (Fig. 2A). For coral species, microbial communities associated with *Porites furcata* and *P. astreoides* were more variable compared to the communities associated with species from the genera *Diploria* and *Acropora*, and the overall spread of coral species was larger compared to coral genus (i.e., coral species microbial community composition are more dissimilar than coral genera). Partial overlaps between sites and times suggest that the spatial and temporal differences in microbial communities were smaller than those observed between distinct coral genera and species (Fig. 2A). These differences were confirmed via PERMANOVA, which showed that coral genus was significantly associated with microbial community structure (PERMANOVA, *F* = 21.73, *R*^2^ = 0.094, *p*-value = 0.001; Table 1). Interestingly, differences in microbial community structure were also significantly associated with coral host species nested within genus, but to a lesser extent than genus (PERMANOVA, *F* = 10.62, *R*^2^ = 0.069*, p*-value = 0.001; Table 1). Overall, this suggests that phylogenetic dissimilarity of coral hosts is positively related to microbiome dissimilarity, with coral host identity at the level of genus acting more strongly on total microbial community differences than at the species level (Table 1).

**Table 1:** PERMANOVA relating the Bray-Curtis dissimilarity in OTU abundance to coral host (species nested within genus, genus), site and time (month).

Abundance Data-(Whole Data)	DF	F	R^2^	p-value
Genus	2	21.73	0.094	0.001
Site [spatial environmental heterogeneity]	3	6.97	0.045	0.001
Time [temporal environmental heterogeneity]	2	9.22	0.040	0.001
Species(Genus)	3	10.62	0.069	0.001
Genus:Site	6	3.91	0.051	0.001
Genus:Time	4	2.04	0.018	0.001
Site:Time [Spatial-Temporal envir. heterogeneity]	6	2.51	0.032	0.001
Species(Genus):Site	6	3.63	0.047	0.001
Species(Genus):Time	6	1.49	0.019	0.001
Genus:Site:Time	12	1.54	0.040	0.001
Species(Genus):Site:Time	12	1.21	0.031	0.004

We next assessed the effects of environmental variation in space (site) on total microbial community structure via PERMANOVA. We found a significant effect of spatial environmental heterogeneity (PERMANOVA, *F* = 6.97, *R*^2^ = 0.045, *p*-value = 0.001), indicating that community composition varied significantly in space and yielded different microbiomes across sites. To detect spatial trends in total microbial community structure, we computed microbial dissimilarity across all pairs of sites for each coral genus. We then used ANCOVA to relate coral genus (factor), distance between sites (covariate), and their interaction to microbial dissimilarity and we computed the statistical significance of each component of the model via Monte Carlo simulations to account for the non-independence of the samples (see methods). This allowed us to determine whether the relationship between total microbial community dissimilarity and distance between sites was consistent across coral genera (Table 2, Fig. 2B). Distance between sites was positively related to microbial dissimilarity across all coral genera (*p-*value = 0.0009), and the mean adjusted microbial dissimilarity significantly differed across coral genera (*p*-value = 0.033). However, the non-significant interaction (*p*-value = 0.728) indicates that the increase in microbial dissimilarity with distance does not differ across coral genera.

**Table 2:** ANCOVA relating the mean Bray-Curtis dissimilarity to distance (site) between coral host (*R*^2^ = 0.541).

	df	F-value	p-value	η^2^
Distance (km)	1	6.065	0.0299	0.232
Genus	2	3.487	0.064	0.267
Distance x Genus	2	0.551	0.590	0.042

Additionally, we assessed whether the total microbial community composition varied over the temporal environment (time) via PERMANOVA. We found a significant effect of temporal environmental heterogeneity (PERMANOVA, *F* = 9.22, *R*^2^ = 0.040, *p*-value = 0.001), which suggests that the whole community composition varied significantly over time. Additionally, we found a significant interaction between space and time (PERMANOVA, *F* = 2.51, *R*^2^ = 0.032, *p*-value = 0.001), indicating that the spatial variation in the total microbiome changed over time (e.g., due to spatiotemporal environmental heterogeneity). However, the effect size of the interaction on microbiome structure, as measured via *R*^2^, was much smaller than those of space and time. Overall, these results suggest that spatial and temporal environmental heterogeneity explain roughly half as much of the variation in the microbiome as host identity operating at both the species and genus levels (spatial + temporal environmental heterogeneity *R*^2^ = 0.085 vs. host identity at genus + species(genus) *R*^2^ = 0.163).

**Figure 3:**
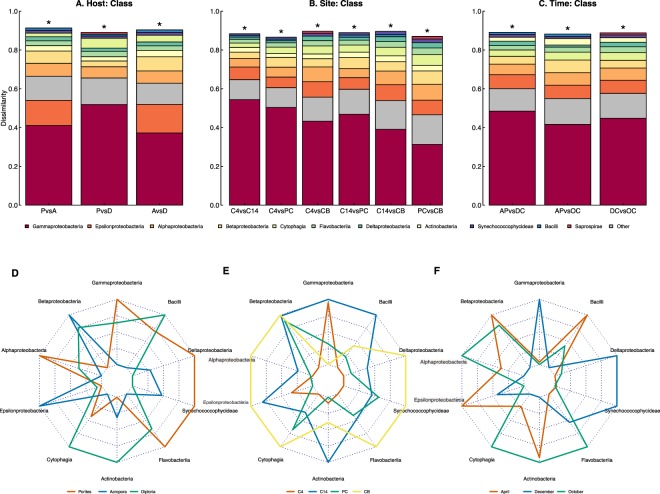
Simper analysis of top 10 represented microbial classes (**A**) Host, (**B**) Site and (**C**) Time along with their associated radar plots comparing microbial community representation for all groups within each factor (**D**) Host, (**E**) Site and (**F**) Time. All pairwise comparisons involving the host are at the level of coral genus. Asterisks indicate significance for within bar comparisons at the α = 0.05 level based on sequential Bonferroni correction.

To determine the top bacterial phylotypes driving the differences in the total microbiome structure between samples across coral genera, sites and times, a similarity percentage analysis (SIMPER) was conducted. All pairwise comparisons between hosts (at genus level), sites, and times were significant at the *α* = 0.05 level following sequential Bonferroni correction (Tables 3–5). Differences in Gammaproteobacteria phylotypes accounted for the largest proportion of dissimilarity across all pairwise comparisons (i.e., between all combinations of genus, site and time; Fig. 3A–C). For coral host, most of the microbial dissimilarity was likewise driven by Gammaproteobacteria, which were more abundant in *Porites* than in *Acropora* and *Diploria* (Fig. 3D). Additionally, we found that Endozoicomonaceae and Campylobacteraceae (class Gammaproteobacteria and Epsilonproteobacteria, respectively) played the largest role in driving the differences in microbiome structure across coral genera (Porites vs Acropora: Endozoicomonaceae - 0.14 and Campylobacteraceae - 0.11; Porites vs Diploria: Pseudomonadaceae - 0.08 and Endozoicomonaceae – 0.07; Acropora vs Diploria: Campylobacteraceae – 0.12 and Endozoicomonaceae - 0.11). For site, Gammaproteobacteria had the highest abundances at Crawl Key 4 (C4) and much lower abundances across all other sites (Fig. 3E). For time, Gammaproteobacteria showed the highest abundances in December and were largely absent in April and October (Fig. 3F). Microbial contribution to community dissimilarity did not change significantly with geographical distance (Supporting Information, Fig. S2). *Porites* and *Diploria* dissimilarity were dominated by Gammaproteobacteria across all distances. Conversely, a more diverse set of microbial classes contributed to dissimilarity in *Acropora*. Overall, the percent contribution of different microbial classes to dissimilarity remained relatively constant across all geographical distances for all coral genera (Supporting Information, Fig. S2).

**Table 3:** Pairwise comparisons of Bray-Curtis dissimilarity between coral genera.

	R^2^	p-value	p-value_adj_
Porites vs Acropora	0.083	0.001	0.003
Porites vs Diploria	0.055	0.001	0.003
Acropora vs Diploria	0.085	0.001	0.003

**Table 4:** Pairwise comparisons of Bray-Curtis dissimilarity between sites.

	R^2^	p-value	p-value_adj_
CK4 vs CK14	0.026	0.001	0.006
CK4 vs PC	0.025	0.001	0.006
CK4 vs CB	0.050	0.001	0.006
CK14 vs PC	0.030	0.001	0.006
CK14 vs CB	0.030	0.001	0.006
PC vs CB	0.022	0.001	0.006

**Table 5:** Pairwise comparisons of Bray-Curtis dissimilarity between times.

	R^2^	p-value	p-value_adj_
April vs December	0.040	0.001	0.003
April vs October	0.015	0.001	0.003
December vs October	0.035	0.001	0.003

### Network analysis

To determine the relative strength of host identity and spatiotemporal environmental heterogeneity on microbial community structure, we computed homophily (heterophily) scores by calculating the average correlation between samples taken from the same (different) coral hosts, sites and times. Coral host had the highest mean homophily score (0.2691), followed by site (0.1883) and time (0.1712). Additionally, coral host had the lowest mean heterophily score (0.0927), followed by site (0.1410) and time (0.1440). This suggests that samples taken from the same coral hosts (genera) had more similar total microbial communities than those taken from the same sites or at the same time. Although these microbial communities appeared to be more similar across samples taken from corals of the same genus than those taken at the same sites and times, the homophily scores were very low in general, suggesting quite a bit of variation across microbiomes.

**Figure 4:**
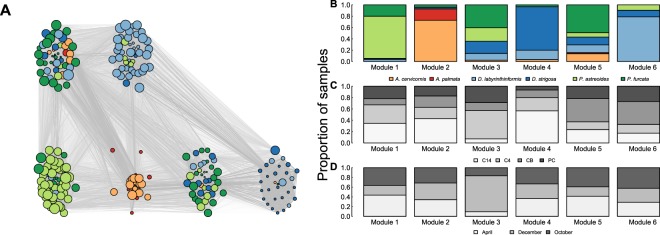
(**A**) Coral microbiome network based on all coral species, sites and times exhibits 6 distinct modules based on microbiome differences across coral samples. (**B**–**D**) Proportion of samples from each (**B**) coral species, (**C**) site and (**D**) time represented in each module. Nodes in the network represent the color-coded coral species each sample is associated with. Node size is proportional to its eigen centrality value.

To determine whether host phylogeny or spatiotemporal environmental heterogeneity could be used to infer microbial community composition, we performed a network analysis across all coral species, genera, sites, and times. In this case, the network exhibited six distinct network modules or clusters of corals (Fig. 4A), with the modularity in this network being significantly larger than that observed in 999 randomly shuffled networks obtained via Monte Carlo simulations (*p*-value = 0.001). Although these network modules were significantly associated with coral species (*p*-value < 2.2 × 10^−16^) and coral genus (*p*-value < 2.2 × 10^−16^), site (*p*-value = 4.6 × 10^−8^) and time (*p*-value = 5.6 × 10^−6^), there was a lot of variation in the predictive power of each of these factors (Table 6).

**Table 6:** χ^2^ analysis relating each factor (species, genus, site and time) to sample module membership.

	df	*χ* ^2^	p-value
Species*	25	536.12	<2.2 × 10^−16^
Genus*	10	376.02	<2.2 × 10^−16^
Site*	15	64.254	4.6 × 10^−8^
Time*	10	42.703	5.6 × 10^−6^

Coral genus was the best predictor of network modularity (correctly classifying 55% of the samples), followed by site (29%) and time (26%). For coral genus, four of the six modules were largely represented by one genus (module 1: *Porites* – 0.95, module 2: *Acropora* – 0.93, module 4: *Diploria* – 0.93, and module 6: *Diploria* – 0.90; Fig. 4B), while modules 3 and 5 were less dominated by one genus (module 3: *Porites* – 0.64 and *Diploria* – 0.33; module 5: *Porites* – 0.57, and *Diploria* – 0.27).

Site and time were lower in their predictive power of network modularity and were both more evenly spread across the six modules than coral genus. For site, the majority (4/6) of the modules included over 10% representation of all sites (Fig. 4C) and all four of these modules had less than 50% representation of each site. Only modules 3 and 4 had at least 50% representation of one site (C4 – 0.50 and C14 – 0.57, respectively). For time, all modules had at minimum 20% representation of each time point, with the exception of module 3 (Fig. 4D). Additionally, it was also the only module that had over 45% representation of one site (module 3: December – 0.74). Therefore, both site and time were well dispersed across all six modules.

Overall, the network analyses suggest that distinct modules largely represent different coral genera regardless of where or when they were sampled. Thus, host microbial composition persists over time and space with coral genus ranking as the most important factor structuring coral microbial communities.

### Eigen centrality analysis

**Figure 5:**
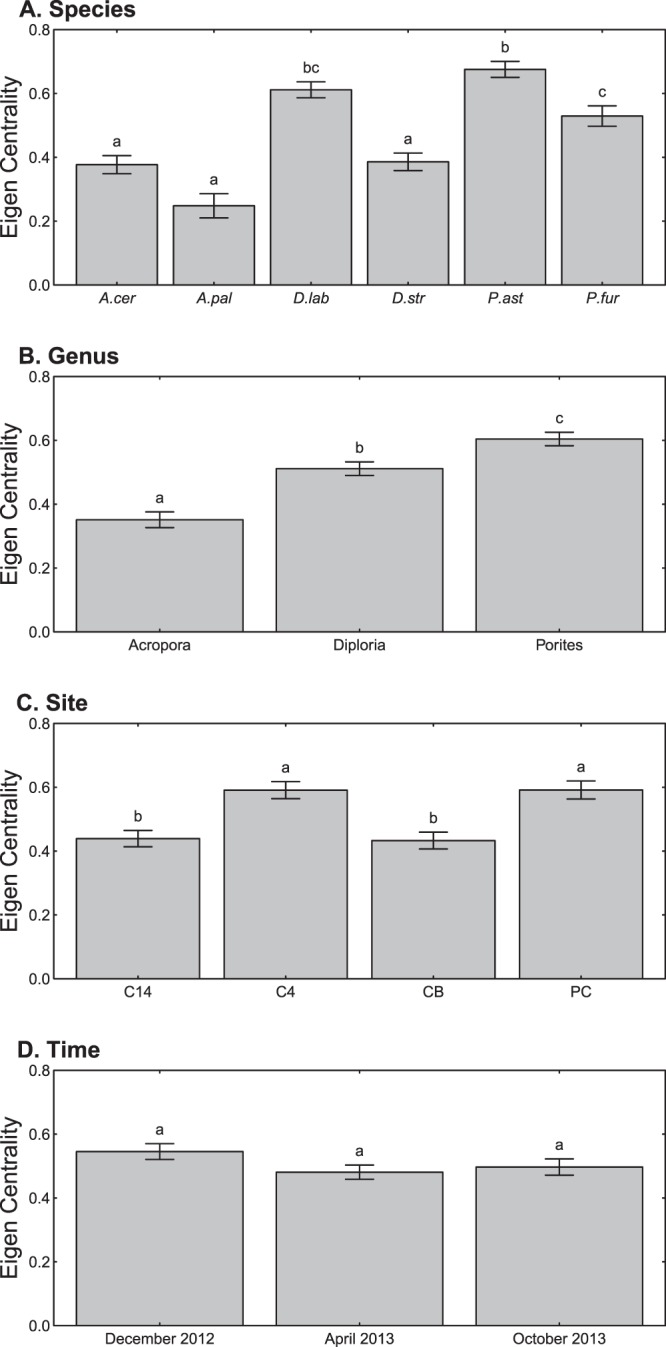
Mean eigencentrality for (**A**) coral species, (**B**) coral genus, (**C**) site, and (**D**) time. Labels indicate which bars are significantly different from one another at the α = 0.05 level based on sequential Bonferroni correction.

To determine the relative influence of host identity and spatiotemporal heterogeneity on total microbial community composition, we computed eigen centrality scores for all nodes in the network. For our network, a connection between two nodes (coral samples) is based on the similarity of their microbiomes as measured via correlation. These similarities (correlations) are calculated between all pairs of nodes in the network. The link connecting pairs of nodes is based on the strength of the correlation in their microbiomes. A high eigen centrality score for a focal node indicates that it is well connected to other nodes in the network that are themselves well connected. Mean eigen centrality scores were then calculated across host-species, host-genus, site and time. For our network, mean eigen centrality was significantly different across genera, but not across space and time (Fig. 5). For genus, there was a linear increase in eigen centrality from *Acropora* (0.351), to *Diploria* (0.511) and *Porites* (0.604). This trend was also observed for mean eigen centrality values across species. Here, eigen centrality is a measure of the strength of host phylogeny, with lower values indicating a higher degree of coral host influence on microbial community structure. Overall, our results suggest that the microbiomes of *Acropora* corals are less well connected to other coral genera than those of *Diploria* or *Porites*.

For site (space), we found that Crawl Key 14 (C14) and CB were significantly different from C4 and Punta Caracol (PC) (Fig. 5C), with C14 and CB having significantly lower eigen centrality means. This suggests that C4 and PC were highly influential in that they shared many of the same microbes commonly found in other samples despite the sites being characterized by different geographical locations (non-protected and protected, respectively) and environmental conditions. Therefore, C4 and PC had a lot in common with most samples, whereas C14 and CB contained more distinct microbial communities.

Finally, mean eigen centrality for the three time points did not differ significantly (Fig. 5D). This suggests that the connectivity of coral microbiome networks remained largely the same throughout the year, even though sampling occurred during typically rainy (December) and dry (October) months. Overall, this suggests that although microbial community composition changed over spatiotemporal scales, the network connectivity remained relatively stable.

## Discussion

Although total microbial community structure varied significantly across coral hosts, space and time, our analyses revealed coral-specific core microbiomes that remained relatively constant in space and time. Thus, as suggested in recent studies^28,30^, focusing on variation in members of the coral-specific microbes rather than the total coral microbiome may promote both the early detection of microbiome perturbations and the identification of pathogenic bacteria linked to disease outbreaks by greatly reducing the number of putative causal agents. Adopting such an approach would effectively reduce both false positives and false negatives by allowing us to ignore natural variation in non-core microbes across spatial and temporal scales in order to focus instead on that of the coral-specific core microbiome. Additionally, our network analyses suggest that systematic variation in the strength of host identity across coral hosts could be used to infer species-specific vulnerability to environmental perturbations and disease outbreaks, and thus inform both conservation and management plans.

### Distinguishing the microbial signal from the noise

The diversity and variability of coral microbial communities have made it difficult to identify the bacteria that play key functional roles. Indeed, our descriptive statistics (at the whole microbial community level) identified high variability in coral microbial communities across coral genus, site (space), and time at broad taxonomic scales (e.g., class). Our multivariate analyses conducted at fine taxonomic scales confirmed these results by revealing that microbial communities varied significantly across coral hosts (genus and species), space and time. However, recent research provides evidence for the persistence of a selective group of bacteria (core microbiome) across environmental gradients^17,42,47^, and across exposure to multiple different stressors^31,48^. Our results are largely consistent with these findings: we found that coral hosts share the same core bacteria across space and time (persistence >95% across all samples within a coral genus) but that core bacterial abundances varied across coral genera. While specific and stable coral microbial interactions have previously been considered unlikely^49^, we suggest that only by sampling across space and time can we establish the necessary baseline information needed to identify core microbial species whose associations with corals remain stable. Doing so would allow us to ignore microbial “noise” in the form of environmentally responsive transient species and focus on the core microbiome “signal” in the form of microbes consistently associated with specific coral species across spatial and temporal scales^28^.

We have shown that coral genera exhibit consistent microbial associations, and a large fraction of the difference between coral host microbiomes can be attributed to a few key microbial classes. Phylotypes representing Gammaproteobacteria and Betaproteobacteria consistently had some of the highest abundances across all coral genera core microbiomes, however, we determined that bacterial phylotypes from Gammaproteobacteria varied across coral genera and similar bacteria were often differentially abundant between coral genera. Additionally, we found that Endozoicomonaceae and Campylobacteraceae (class Gammaproteobacteria and Epsilonproteobacteria, respectively) played the largest role in driving the differences in microbiome structure across coral genera, while phylotypes from Betaproteobacteria accounted for much less. This suggests that phylotypes from Betaproteobacteria are to some extent consistent across coral genera. Altogether, even though many of the microbial species were shared among most coral hosts, we were able to identify microbial phylotypes that were more abundant on a subset of those coral hosts and this association persisted in space and time. This indicates that some members of the microbial community demonstrate a significant level of specificity across coral hosts and potentially facilitate the success of their coral host in diverse environments. Therefore, focusing on variation within these coral-specific core bacteria, which represent members of the microbial community likely to be the most functionally significant to their host, could provide clues about the drivers of community persistence in space and time in the face of environmental perturbations.

### Abiotic and biotic drivers of microbiome structure

Although coral host identity is a stronger driver of the microbial community structure compared to spatiotemporal environmental heterogeneity and dispersal, we found significant spatiotemporal heterogeneity in the total microbial community composition, which suggests that community structure varies across temporal and spatial scales. Similarly, previous studies have shown that microbial community structure varies spatially due to habitat differences between sites^27,47^. This is potentially surprising because marine populations have historically been assumed to be relatively well mixed because of strong oceanographic currents^50^. Such broad-scale dispersal has the potential to spatially homogenize populations by eroding the effects of finer-scale variation in environmental conditions and habitat structure on abundance. However, recent research has demonstrated that self-recruitment or self-seeding are high in some marine organisms, with propagules returning to their natal sites^51,52^. Even fish species that produce pelagic propagules that spend weeks in the water column and thus have the potential to be transported over large distances typically disperse less than 14 km from their natal sites^53,54^.

Our study contributes to this body of work by showing that despite the existence of stable coral-specific microbial associations, total microbial community dissimilarity increases with geographical distance (Fig. 2B). Hence, the farther apart coral hosts are, the more their overall microbial communities will differ. These results thus suggest that dispersal is not sufficiently strong to suppress site-to-site variation in microbiome structure, even in a relatively environmentally homogeneous region measuring less than 30 kilometers. Detecting signatures of spatial structure in such a small region suggests that regional processes such as dispersal limitation and environmental heterogeneity play a role in structuring coral microbiomes at fine spatial scales. Naturally, the relative importance of factors like space and time is likely to rise when samples are obtained across larger spatial and temporal scales. Hence, identifying the temporal and spatial scales at which coral-specific core microbiomes emerge is a critical next step in order to better understand the relative importance of their local vs. regional biotic and abiotic drivers.

### The network structure of the microbiome

Network approaches have been successfully applied to study bacteria-bacteria^55^, phage-bacteria^56^, bacteria-eukaryote networks in sequence data^57^, and on a coral-microbial core microbiome^28^. Utilizing network theory, we identified a hidden structure within the coral associated microbial communities. We found the microbial networks were significantly compartmentalized and strongly clustered based on coral host identity. Hence, the phylogenetic identity of the coral host appears to largely control microbiome structure across space and time. Furthermore, we found that this type of phylogenetic signal in coral microbiomes varies significantly across coral genera and species. Specifically, *Acropora* contain the least amount of connections to heterospecific hosts, exhibiting the most individualistic microbiome. Conversely, *Porites* are involved with the most connections to heterospecifics, and contain some of the most influential nodes in the network. This suggests that coral hosts with weakly connected microbiomes, are potentially more robust to perturbations of their microbiomes compared to highly connected coral hosts. Interestingly, this trend aligns with the dispersion in the ordination (Fig. 2A). Within this figure, *Porites* species has the highest variation in microbial communities (indicating a more generalist composition), while *Acropora* shows the least community variation. It has been posited that hosts can actively select for their microbial communities through two processes, one such mechanism being the sanctioning of non-beneficial microbes via antibiotics^34^. In addition, different corals may provide different host-derived nutrients and niches to colonizing bacteria^58^, offering more resources to beneficial bacteria to promote growth. Although our data suggests that coral host identity plays a role in the composition of the microbial community, we are currently unable to determine which mechanism is governing these communities. However, our network analysis results do suggest that studying the stability of the microbiome structure in space and time can reveal the degree to which they are susceptible to perturbations such as diseases and environmental stressors.

## Conclusion

Microbial symbioses with coral reefs have been increasingly recognized as important contributors to their ability to ward-off diseases and combat environmental perturbations^27,59,60^. Although much of the research to date has focused on disturbed coral microbiomes, understanding the variation in the structure of healthy coral microbiomes will provide critical insight into the stability of this complex ecological association. The results presented here suggest that local processes such as host phylosymbiosis play a more important role than regional dispersal or environmental heterogeneity in dictating the structure and persistence of microbial communities across spatiotemporal scales. Additionally, we showed that the strength of host identity varies across coral genera, which suggests that some corals are likely more robust to environmental perturbations. Finally, our results highlight the importance of characterizing the core coral microbiome and the critical spatiotemporal scales at which it emerges. Developing this kind of baseline information regarding the normal functioning of the coral microbiome will allow us to identify the abiotic and biotic drivers of microbiome composition, and thus improve our ability to both diagnose perturbed corals and discover the bacterial origins of emerging coral diseases.

## Methods

### Data acquisition

Data were collected and processed by Chu & Vollmer^18^. We summarize their methods below but a full and detailed description of their approach can be found in Chu & Vollmer (2016). Coral tissue samples were collected from 100 tagged coral colonies near Bocas del Toro, Panama, in December 2012, April 2013, and October 2013 (See Supplementary Information: Table S1). All coral colonies sampled throughout the time points were in a healthy state (i.e., no signs of disease). Bacterial communities have been found to vary significantly in terms of coral colony size (age brackets)^61^, but remain consistent between replicates within the same size class, therefore, all corals sampled were of approximately the same size within a coral species across all sites. Each of the 100 tagged coral colonies was repeatedly sampled across time points on one of four reefs: two protected inshore reefs, Punta Caracol (PC; N9.37804 N, W82.30335) and Casa Blanca (CB; N9.36028, W82.27760), and two exposed offshore reefs, Crawl Key 14 (C14; N9.25398, W82.12595) and Crawl Key 4 (C4; N9.25862, W82.12708). Samples were kept in a cooler at ambient seawater temperature and immediately transported back to the lab. Samples were then preserved in CHAOS buffer (4 M guanidine thiocyanate, 0.5% N-laurosil-sarcosine, 25 mM Tris pH 8.0, 0.1 M 2-mercapto ethanol), allowed to lyse for one week at room temperature, and stored at −20 °C until further processing.

DNA from preserved coral samples were extracted using an Agencourt DNAdvance kit, in order to prepare 16S rDNA profiling libraries. 16S libraries of the hypervariable V6 region were then prepared using a two-step PCR protocol and combinatorial barcodes outlined in Gloor *et al*.^62^, resulting in amplicon lengths from ~100–120 bp. 16S sequencing data were clustered into operational taxonomic units (OTUs) using a 97% identity threshold in QIIME^63^. Default QIIME settings were used to align reads^64^, to remove chimera sequences^65^, and to assign taxonomy^66,67^.

### Data analysis

#### Multivariate analyses

Normalized abundances of OTUs were computed using the DESeq2 package^68^ in order to correct for library size. All analyses utilized the full dataset (i.e., no filtering of rare OTUs). To test for differences among bacterial communities between hosts (genus and species), sites and times, a PERMANOVA was performed using Bray-Curtis dissimilarity with the vegan package in R^69^ that tested the independent and joint effects of host, site and time on microbial community structure. Post-hoc pairwise comparisons with sequential Bonferroni correction were performed to determine the differences in microbial community structure between levels within each factor (e.g., compare sites or times). To visualize the PERMANOVA results, we conducted a non-metric Multidimensional Scaling analysis (nMDS) of the bacterial community composition based on Bray-Curtis dissimilarity using the vegan package. We also constructed 95% confidence ellipses for hosts, sites, and times in order to highlight significant differences in microbial community structure. Additionally, an analysis of covariance (ANCOVA) was conducted to determine whether microbial community Bray-Curtis dissimilarity differed across coral hosts (factor) and/or was related to geographical distance between sites (covariate). To abide by the assumptions of linearity, both Bray-Curtis dissimilarity and geographical distance were log-transformed. Due to non-independence between samples (spatial and temporal autocorrelation), we used Monte Carlo simulations to determine whether coral genus, distance and distance by dissimilarity observed in our data were significant. Specifically, we randomly shuffled the distances and genera 999 times in order to scramble the association between distance, genus and dissimilarity. We then conducted an ANCOVA on each of the 999 shuffled datasets and extracted the *F* statistic for each model component. We then calculated the *p*-value as the proportion of the 999 shuffled datasets that produced an *F* statistic that was greater than or equal to the one observed in the original data. Utilizing the vegan package in R, we then conducted similarity percentage analysis (SIMPER)^70^ to compare pairs of microbial communities across coral samples (e.g., *Porites* microbial community vs. *Acropora* microbial community) and determine the main microbial classes driving any differences. To do this, we conducted the analyses as the OTU level and then aggregated (summed) the results at the taxonomic level of class. Radar plots were constructed to visualize the microbial class differences for each group within all three factors.

#### Network analysis

We performed multiple network analyses on the coral-microbiome network using the igraph package in R^71^. We began by using the coral-microbiome data to build a correlation matrix using the Pearson product-moment correlation. Specifically, we measured the correlation between the microbial communities of each coral sample pair. To assess how the strength of host phylosymbiosis varied across genera in space and time, we computed homophily and heterophily scores using the correlation matrix for each coral genus to determine the degree to which they tend to share a common microbiome with conspecifics vs. heterospecifics. Additionally, we computed homophily and heterophily scores for site and time to determine the relative strength of host identity and the spatiotemporal environmental heterogeneity on the microbial community structure.

We then used this correlation matrix to create a weighted and undirected graph, where the weight of the edges or links between coral hosts represent the shared microbial species between the hosts and their similarities (correlations) in abundances. We then used a community detection algorithm based on the leading eigen vector centrality^72^ to document the degree of compartmentalization by identifying distinct clusters (modules) of coral hosts in the network (i.e., groups of hosts that were more connected to each other than to others). Additionally, we used Monte Carlo simulations to determine whether the degree of compartmentalization or modularity observed in the network was significant. Specifically, we generated 999 random networks by shuffling the correlations within each coral host and then computed modularity. We then calculated the *p*-value as the proportion of random networks whose modularity was greater than or equal to that observed in the original (non-shuffled) network.

A question of interest was whether the distinct modules identified through the network analysis were associated with differences between coral hosts (genus or species), reef sites and months. We thus conducted a χ^2^ test to determine whether there was an association between coral host (genus or species), reef site, and month and their assigned module obtained via the network community detection algorithm. We then calculated the misclassification rate for coral host (genus), site, and time to determine the rate at which each factor incorrectly classified samples into a module.

We were particularly interested in whether we could determine the degree of host identity between hosts (at the level of genus and species). To do so, we used an algorithm that finds the eigenvector centrality scores for each node within the network^71,73^. Eigenvector centrality scores arise from a reciprocal process in which the centrality of each node (i.e., coral host) is proportional to the sum of the centralities of those nodes with which they are connected to. In general, nodes with high eigenvector centralities are connected to many other nodes which are, in turn, connected to many others. Here, this implies that the largest values will be obtained by coral hosts in large (or high-density) clusters. Specifically, the higher the value, the more similar the coral’s microbiome is to that of others, which indicates the coral phylogenetic signal is weak. Alternatively, the lower the value for a host, the more distinct their microbiome, which suggests a higher degree of host phylosymbiosis.

## Supplementary information

Supplementary Information
