# Supplementary material for: Structure and stability of the coral microbiome in space and time
Source: Sci Rep. 2019 May 1;9:6785. doi: 10.1038/s41598-019-43268-6 (PMC6494856; doi:10.1038/s41598-019-43268-6)
Supplement: Supplementary file 1 — Supplementary Information [file 41598_2019_43268_MOESM1_ESM.docx]

# Structure and stability of the coral microbiome in space and time

Courtney M. Dunphy, Tarik C. Gouhier, Nathaniel D. Chu and Steven V. Vollmer

**Supporting Information**

**Table S1:** Sample information across coral genera, site and time

|  | Acropora (*n = 75*) | Porites (*n = 117*) | Diploria (*n = 118*) |
| --- | --- | --- | --- |
| April *(n = 99*) | *(n = 25)* | *(n = 38)* | *(n = 36)* |
| C4 | 5 | 10 | 10 |
| C14 | 10 | 10 | 10 |
| PC | 5 | 8 | 7 |
| CB | 5 | 10 | 9 |
| December (*n = 100*) | *(n = 25)* | *(n = 39)* | *(n = 36)* |
| C4 | 5 | 10 | 10 |
| C14 | 10 | 10 | 10 |
| PC | 5 | 9 | 7 |
| CB | 5 | 10 | 9 |
| October (*n = 101*) | *(n = 25)* | *(n = 40)* | *(n = 36)* |
| C4 | 5 | 10 | 10 |
| C14 | 10 | 10 | 10 |
| PC | 5 | 10 | 7 |
| CB | 5 | 10 | 9 |

**Figure S1**: Simper analysis of top 10 represented microbial families for all three coral genera

**Figure S2**: Simper analysis of all pairwise comparisons for most represented microbial classes contribution to dissimilarity for all three coral genera across all site distances

**Figure S1**

**Figure S2**
